# Supplementary material for: Food insecurity and hypertension: A systematic review and meta-analysis
Source: PLoS One. 2020 Nov 17;15(11):e0241628. doi: 10.1371/journal.pone.0241628 (PMC7671545; doi:10.1371/journal.pone.0241628)
Supplement: S1 File — (PDF) [file pone.0241628.s001.pdf]

Food Insecurity and Hypertension: A Systematic Review and Meta-analysis

S1 Supplemental Information

Further elaboration on the AXIS Delphi tool for assessment of study quality for all studies included for review and meta-analysis.

Two authors independently reviewed all 36 manuscripts included in this study using the AXIS Delphi tool for quality assessment of cross-sectional studies [1]. While evaluation by the AXIS tool did not yield results that warranted the removal of any included study from this review, several points are worth mentioning. First, all 36 studies were deemed satisfactory for 15 out of the 20 measures included in the AXIS tool. 2 of the remaining 5 measures concerned categorization of non-responders in the collection of survey data, for which 12 studies did not meet criteria. An additional 30 studies were found not to justify their sample sizes in the manuscript text. However, neither of these findings were thought to be sufficiently concerning to remove these studies as it is not currently universal practice to report data on survey non-responders or justify sample sizes. Furthermore, a relatively low percentage of studies doing so has been reported in previous systematic reviews by the authors and others [2-6]. For one of the final two measures for which there were not 100% satisfactory findings, 2 studies were found to not clearly define statistical significance in their findings. However, this also was not determined to be sufficient to remove these studies from meta-analysis as the effect sizes of these studies were calculated directly from the studies' primary data thereby yielding helpful data for the purpose of this study regardless of the original study's decision to not clearly define statistical significance. Finally, in the last measure for which there were not 100% satisfactory findings, 6 studies were found not to clearly discuss limitations. Although this is a concerning finding, it was determined that an absence of clearly defined limitations was not sufficient to discredit the primary data extracted from those studies as the studies had otherwise met satisfactory criteria for study quality.

REFERENCES

1. Downes MJ, Brennan ML, Williams HC, Dean RS. Development of a critical appraisal tool to assess the quality of cross-sectional studies (AXIS). BMJ Open. 2016;6(12):e011458.

2. Arenas D, Beltran S, Zhou S, Goldberg L. Cocaine, cardiomyopathy, and heart failure: A systematic review of clinical studies and meta-analysis of effect sizes. In: APHA's 2019 Annual Meeting and Expo (Nov 2-Nov 6). American Public Health Association; 2019.

3. Arenas DJ, Thomas A, Wang J, DeLisser HM. A systematic review and meta-analysis of depression, anxiety, and sleep disorders in US adults with food insecurity. J Gen Intern Med. 2019;1–9.

4. Brink Y, Louw QA. A systematic review of the relationship between sitting and upper quadrant musculoskeletal pain in children and adolescents. Man Ther. 2013;18(4):281–288.

5. Basma B, Savage R. Teacher professional development and student literacy growth: A systematic review and meta-analysis. Springer; 2018.

6. Douville F, Godin G, Vézina-Im L-A. Organ and tissue donation in clinical settings: a systematic review of the impact of interventions aimed at health professionals. Transplant Res. 2014;3(1):8.
