# Supplementary material for: Food insecurity and hypertension: A systematic review and meta-analysis
Source: PLoS One. 2020 Nov 17;15(11):e0241628. doi: 10.1371/journal.pone.0241628 (PMC7671545; doi:10.1371/journal.pone.0241628)
Supplement: S2 File — (PDF) [file pone.0241628.s002.pdf]

Food insecurity and hypertension: A systematic review and meta-analysis

S2 Supplemental Information

Figure S2.1. Inter-rater agreement in abstract exclusion.
Group agreement in the inclusion/exclusion of abstracts is shown by the upper and lower limits of Fleiss kappa (green), the group inter-rater metric. A Fleiss kappa of 0.68 [0.66, 0.71] signifies inter-rater agreement much higher than expected from random chance. All possible permutations of inter-rater agreement between all users (i.e. 0 and 3, or 1 and 2) are also shown in blue. Orange denotes the average of the permutations and standard deviation. This graph serves to show that not only was the group agreement high but also all individual raters had high agreement with the others. Only abstracts excluded by all 4 users were automatically discarded, otherwise they were text evaluated. (For more details on the methodology, see Arenas DJ. Inter-Rater: Software for analysis of inter-rater reliability by permutating pairs of multiple users. ArXiv Prepr ArXiv180905731. 2018).

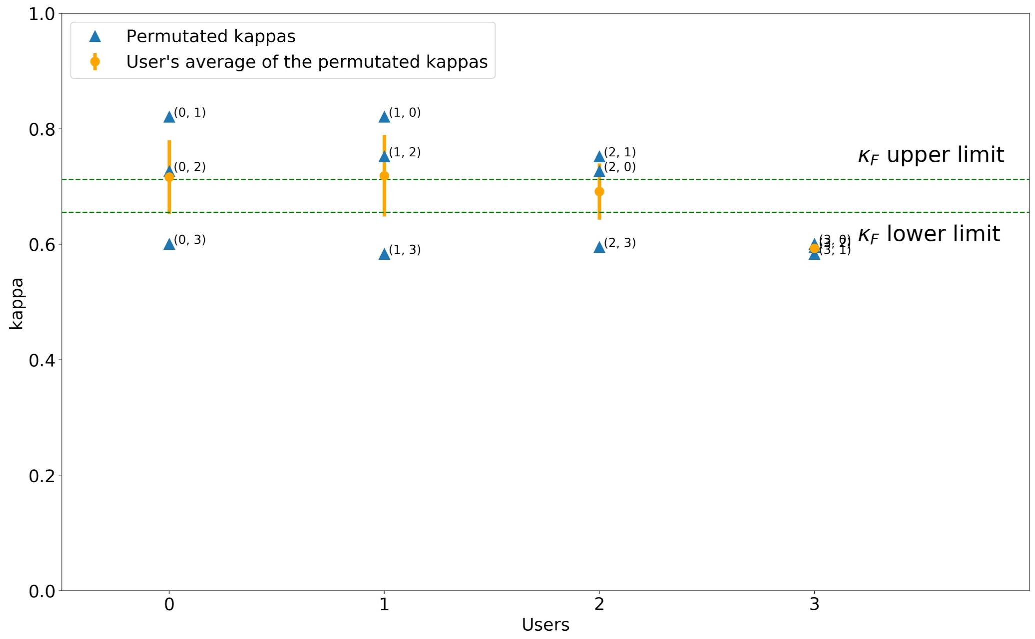

Table S2.1. All 21 adult studies which measured or produced odds ratios for food insecurity and hypertension.

Study (year)	Target population	Sample size	HTN measurement	Results (* = calculated from primary data)
Weigel2007(1)	USA. Households of migrant and seasonal farmworkers from Mexico	100	Measured by researchers. Systolic > 130 or Diastolic > 90	OR = 0.66 [0.23, 1.91]*
Terrell2009(2)	USA. General population. NHANES 1999-2004	15199	Self-reported hypertension diagnosis	AOR = 1.13 [0.91, 1.39]
Perez-Escamilla2014(3)	Mexico. General Population.	55335	Self-reported hypertension diagnosis	OR = 1.14 [1.08, 1.19] *
Berkowitz2013(4)	USA. Diabetic adults. NHANES 1999-2008	2557	Measured by researchers. Cutoffs: >140/>90	AOR = 1.10 [0.75, 1.61]
Ford2013(5)	USA. General population. NHANES 2003-2008	10455	Mixed. BP measurements or medication history	OR = 0.87 [0.78, 0.96]*
Shariff2014(6)	Malaysia. Low-income women.	625	Measured by researchers. BP>130/85	AOR = 0.99 [0.54, 1.50]
Sattler2014(7)	Georgia (USA) Advance POMP 6 Study	4731	Self-reported hypertension diagnosis	OR = 1.08 [0.63-1.84]*
Berkowitz2015(8)	USA Diabetic patients	411	Medical Records. BP>140/90	AOR= 1.58 (0.66 – 3.76)
Moreno2015(9)	USA. Diabetic Latinos..	250	Medical Records. BP>140/90	AOR = 1.02 [0.50, 2.09]
Wang2015(10)	USA. Veterans. VACS 2002-2008	6709	Measured by researchers with cutoff: BP > 140/90	AOR = 1.59 [1.17, 2.17]
Shiue2016(11)	USA. General population. NHANES 2005-2006	4924	Measured by researchers with cutoff: BP > 140/90.	OR = 1.26 [0.80-1.99]*

Banerjee2017(12)	USA. Adults with and without CKD. NHANES 1988-1994	12768	Mixed. Self-report, measured or medication history.	OR = 1.03 [0.63, 1.70] *
Berkowitz2017(13)	USA. General population. NHANES 2005-2010.	21196	Measured by researchers: BP>140/90	OR = 0.80 [0.73, 0.88] *
Weigel2019(14)	USA. Mexican immigrants at border (El Paso, TX)	75	Measured by researchers. BP > 130/80.	AOR= 0.98 [0.72, 1.36]
Mendy2018(15)	USA. General population. 2015 Behavioral Risk Factor Surveillance System.	5870	Self-reported hypertension diagnosis	AOR= 1.51 [1.21, 1.88]
Dong2018(16)	USA. Adults on probation.	304	Measured by researchers: BP>140/90	OR = 0.92 [0.53, 1.60]*
Garcia2018(17)	USA. General Population. NHIS (2011-2014) and MEP (2013-2015)	14879	Self-reported hypertension diagnosis.	OR = 1.45 [1.32, 1.59]*
Helmick2020(18)	USA. Dan River Region. DRPHC Surveillance Survey	930	Self-reported hypertension diagnosis	OR = 1.70 [1.27, 2.47]
Blue Bird2017(19)	Tribal participants	513	Self-reported hypertension diagnosis	OR=1.65 [1.15, 2.35]*
Venci2018(20)	USA. General population. NHANES 2011	30010	Self-reported hypertension diagnosis	OR: 2.20 [2.05, 2.36]*
Schroeder2019(21)	USA. Diabetics 65 and up. Kaiser PCO 2012-2016.	2226	Mixed: EMR diagnosis and BP cutoff BP>140/90	OR = 1.08 [0.82, 1.43]*

Table S2.2. All 7 adult studies which produced hedges g (SMD) for food insecurity and blood pressure.**

Study	Target population	Sample size	HTN measurement	Results (* = calculated from primary data)
Ford2013(5)	USA. General population. NHANES 2003-2008	10455	Measured by researchers (systolic pressure)	g = 0.00 [-0.04, 0.05]*
Moghadam2016(22)	Iran. Diabetic patients	243	Measured by researchers (systolic pressure)	g = 0.14 [-0.13, 0.41]*
Weigel2016(23)	Ecuador. Women. Low-income	269	Measured by researchers (systolic pressure)	g = 0.05 [-0.24, 0.34]*
Shalowitz2017(24)	USA. Illinois.	336	Measured by researchers (systolic pressure)	g = -0.03 [-0.25, 0.19]*
Faramarzi2019(25)	Iran Cohort Study	151	Measured by researchers (systolic pressure)	g = -0.48 [-1.10, 0.14]*
Holben2006(26)	Rural Appalachian	802	Measured by researchers (diastolic pressure)	g = -0.10 [-0.07, 0.26]*
Parker2010(27)	USA. General population. NHANES 1999-2006	6138	Measured by researchers (diastolic pressure)	g = -0.02 [-0.08, 0.04]*
Moghadam2016(22)	Iran. Diabetic patients	243	Measured by researchers (diastolic pressure)	g = 0.37 [0.10, 0.64]*
Weigel2016(23)	Ecuador. Women. Low-income.	269	Measured by researchers (diastolic pressure)	g = 0.13 [-0.16, 0.42]*
Shalowitz2017(24)	USA. Illinois.	336	Measured by researchers (diastolic pressure)	g = -0.10 [-0.32, 0.11]*
Mahmoodi2017(28) ***	Iran. General population. Kerman Coronary Artery Disease Risk Study	231	Measured by researchers (diastolic pressure)	g = -0.11 [-0.39, 0.16]*
Faramarzi2019(25)	Iran Cohort Study	151	Measured by researchers (diastolic pressure)	g = -0.26 [-0.60, 0.08]*

**(Studies are repeated which contained data for both systolic and diastolic blood pressure analyzed separately)

***(Mahmoodi 2017 was ultimately excluded from the adult diastolic SMD meta-analysis as it contained undifferentiated data involving pediatric and adult patients)d

Table S2.3. All five pediatric studies which measured or produced odds ratios for food insecurity and hypertension.

Study (year)	Target population	Sample size	HTN measurement	Results (* = calculated from primary data)
Holben2015(29)	USA. Ages 12-18 years old. NHANES 1999-2006.	7435	Measured by researchers	AOR = 1.07 [0.74, 1.55]*
Bahadur2018(30)	USA. Patients in Federally Qualified Health Center in New Jersey	486	Inspection of medical records	OR = 1.05 [0.05. 22]*
Rongstad2018(31)	USA. Ages 0-20. Madison, Wisconsin	1330	Inspection of medical records	OR = 8.51 [0.40, 181]*
South2019(32)	USA. Ages 8-17. NHANES 2007-2014	7125	Measured by researchers or previous diagnosis or medication history	AOR = 1.42 [1.03 - 1.96]
Lee2019(33)	USA. Ages 12-19. NHANES 2003-2014	2662	Mixed. If BP exceeded 90th percentile for age, sex and height. OR medication history	AOR = 1.65 [1.38, 1.98]

Table S2.4. All three studies which measured food insecurity and hypertension and did not differentiate data by adult and pediatric subjects.

Study (year)	Target population	Sample size	HTN measurement	Results
Vozoris2003(34)	Canada. National Population Health Survey 1996-1997	3204	Self-reported diagnosis of "High Blood Pressure"	AOR = 1.6 [1.2, 2.1]
Mahmoodi2017(28)	Iran. Diabetic patients 15-75 years old. Kerman coronary artery disease risk study.	520	Measured by researchers. Cutoff: BP > 140/90	AOR = 0.99 [0.98, 1.00]
Endeweld2018(35)	Israel. Families receiving food support. National Food Security Program.	362	Self-report of previous hypertension diagnosis	OR= 3.06

REFERENCES

1. Weigel MM, Armijos RX, Hall YP, Ramirez Y, Orozco R. The household food insecurity and health outcomes of US–Mexico border migrant and seasonal farmworkers. J Immigr Minor Health. 2007;9(3):157–169.

2. Terrell A. Is food insecurity associated with chronic disease and chronic disease control? Ethn Dis. 2009;19(2 suppl. 3).

3. Pérez-Escamilla R, Villalpando S, Shamah-Levy T, Méndez-Gómez Humarán I. Household food insecurity, diabetes and hypertension among Mexican adults: Results from Ensanut 2012. Salud Pública México. 2014;56:s62–s70.

4. Berkowitz SA, Baggett TP, Wexler DJ, Huskey KW, Wee CC. Food insecurity and metabolic control among US adults with diabetes. Diabetes Care. 2013;36(10):3093–3099.

5. Ford ES. Food security and cardiovascular disease risk among adults in the United States: findings from the National Health and Nutrition Examination Survey, 2003-2008. Prev Chronic Dis. 2013;10:E202–E202.

6. Shariff ZM, Sulaiman N, Jalil RA, Yen WC, Yaw YH, Mohd Taib MN, et al. Food insecurity and the metabolic syndrome among women from low income communities in Malaysia. Asia Pac J Clin Nutr. 2014;23(1):138.

7. Sattler ELP, Lee JS, Bhargava V. Food insecurity and medication adherence in low-income older Medicare beneficiaries with type 2 diabetes. J Nutr Gerontol Geriatr. 2014;33(4):401–417.

8. Berkowitz SA, Meigs JB, DeWalt D, Seligman HK, Barnard LS, Bright O-JM, et al. Material need insecurities, diabetes control, and care utilization: Results from the Measuring Economic iNsecurity in Diabetes (MEND) study. JAMA Intern Med. 2015;175(2):257.

9. Moreno G, Morales LS, Isiordia M, de Jaimes FN, Tseng C-H, Noguera C, et al. Latinos with diabetes and food insecurity in an agricultural community. Med Care. 2015;53(5):423.

10. Wang EA, McGinnis KA, Goulet J, Bryant K, Gibert C, Leaf DA, et al. Food insecurity and health: data from the Veterans Aging Cohort Study. Public Health Rep. 2015;130(3):261–268.

11. Shiue I. People with diabetes, respiratory, liver or mental disorders, higher urinary antimony, bisphenol A, or pesticides had higher food insecurity: USA NHANES, 2005–2006. Environ Sci Pollut Res. 2016;23(1):198–205.

12. Banerjee T, Crews DC, Wesson DE, Dharmarajan S, Saran R, Burrows NR, et al. Food insecurity, CKD, and subsequent ESRD in US adults. Am J Kidney Dis. 2017;70(1):38–47.

13. Berkowitz SA, Berkowitz TS, Meigs JB, Wexler DJ. Trends in food insecurity for adults

with cardiometabolic disease in the United States: 2005-2012. PloS One. 2017;12(6).

14. Weigel MM, Armijos RX. Food insecurity, Cardiometabolic health, and health care in US-Mexico border immigrant adults: An exploratory study. J Immigr Minor Health. 2019;21(5):1085–1094.

15. Mendy VL, Vargas R, Cannon-Smith G, Payton M, Enkhmaa B, Zhang L. Food insecurity and cardiovascular disease risk factors among Mississippi adults. Int J Environ Res Public Health. 2018;15(9):2016.

16. Dong KR, Must A, Tang AM, Stopka TJ, Beckwith CG. Food insecurity, morbidities, and substance use in adults on probation in Rhode Island. J Urban Health. 2018;95(4):564–575.

17. Garcia SP, Haddix A, Barnett K. Peer Reviewed: Incremental Health Care Costs Associated With Food Insecurity and Chronic Conditions Among Older Adults. Prev Chronic Dis. 2018;15.

18. Helmick M, Smith TM, Parks CA, Hill JL. Food insecurity increases odds of diabetes and hypertension, not obesity in medically underserved region. J Hunger Environ Nutr. 2020;15(1):128–139.

19. Blue Bird Jernigan V, Wetherill MS, Hearod J, Jacob T, Salvatore AL, Cannady T, et al. Food insecurity and chronic diseases among American Indians in rural Oklahoma: The THRIVE study. Am J Public Health. 2017;107(3):441–446.

20. Venci BJ, Lee S-Y. Functional limitation and chronic diseases are associated with food insecurity among US adults. Ann Epidemiol. 2018;28(3):182–188.

21. Schroeder EB, Zeng C, Sterrett AT, Kimpo TK, Paolino AR, Steiner JF. The longitudinal relationship between food insecurity in older adults with diabetes and emergency department visits, hospitalizations, hemoglobin A1c, and medication adherence. J Diabetes Complications. 2019;33(4):289–295.

22. Moghadam SAHZ, Javadi M, Mohammadpooral A. Relationship between Food Security with Sugar Level and Blood Pressure in Diabetes Type 2 in Tehran. Electron Physician. 2016;8(12):3398.

23. Weigel MM, Armijos RX, Racines M, Cevallos W, Castro NP. Association of household food insecurity with the mental and physical health of low-income urban Ecuadorian women with children. J Environ Public Health. 2016;2016.

24. Shalowitz MU, Eng JS, McKinney CO, Krohn J, Lapin B, Wang CH, et al. Food security is related to adult type 2 diabetes control over time in a United States safety net primary care clinic population. Nutr Diabetes. 2017;7(5):e277–e277.

25. Faramarzi E, Somi M, Ostadrahimi A, Dastgiri S, Nahand MG, Jafarabadi MA, et al. Association between food insecurity and metabolic syndrome in North West of Iran: Azar Cohort study. J Cardiovasc Thorac Res. 2019;11(3):196.

26. Holben DH, Pheley AM. Peer reviewed: Diabetes risk and obesity in food-insecure households in rural Appalachian Ohio. Prev Chronic Dis. 2006;3(3).

27. Parker ED, Widome R, Nettleton JA, Pereira MA. Food security and metabolic syndrome in US adults and adolescents: findings from the National Health and Nutrition Examination Survey, 1999–2006. Ann Epidemiol. 2010;20(5):364–370.

28. Mahmoodi MR, Najafipour H, Mohsenpour MA, Amiri M. The relationship between food insecurity with cardiovascular risk markers and metabolic syndrome components in patients with diabetes: A population-based study from Kerman coronary artery disease risk study. J Res Med Sci Off J Isfahan Univ Med Sci. 2017;22.

29. Holben DH, Taylor CA. Food insecurity and its association with central obesity and other markers of metabolic syndrome among persons aged 12 to 18 years in the United States. J Am Osteopath Assoc. 2015;115(9):536–43.

30. Bahadur K, Pai S, Thoby E, Petrova A. Frequency of food insecurity and associated health outcomes in pediatric patients at a federally qualified health center. J Community Health. 2018;43(5):896–900.

31. Rongstad R, Neuman M, Pillai P, Birstler J, Hanrahan L. Screening Pediatric Patients for Food Insecurity: A Retrospective Cross-Sectional Study of. WMJ. 2018;122.

32. South AM, Palakshappa D, Brown CL. Relationship between food insecurity and high blood pressure in a national sample of children and adolescents. Pediatr Nephrol. 2019;34(9):1583–1590.

33. Lee AM, Scharf RJ, Filipp SL, Gurka MJ, DeBoer MD. Food Insecurity Is Associated with Prediabetes Risk Among US Adolescents, NHANES 2003–2014. Metab Syndr Relat Disord. 2019;17(7):347–354.

34. Vozoris NT, Tarasuk VS. Household food insufficiency is associated with poorer health. J Nutr. 2003;133(1):120–126.

35. Endeweld M, Goldsmith R, Endevelt R. The demographic and morbidity characteristics of a population receiving food support in Israel. Isr J Health Policy Res. 2018;7(1):54.
