# Supplementary material for: Food insecurity and hypertension: A systematic review and meta-analysis
Source: PLoS One. 2020 Nov 17;15(11):e0241628. doi: 10.1371/journal.pone.0241628 (PMC7671545; doi:10.1371/journal.pone.0241628)

# Food Insecurity and Hypertension: A Systematic Review and Meta-analysis

## S3 Supplemental Information

**Fig S3.1. Meta-analysis of odds ratio studies which determined hypertension diagnoses by electronic health record inspection.**

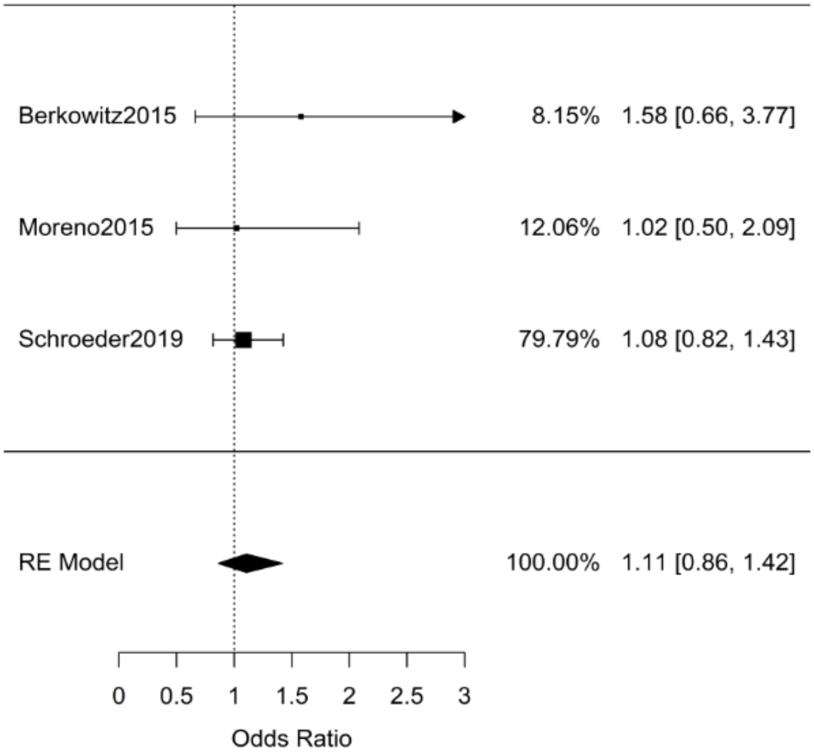

**Fig S3.2. Meta-analysis of odds ratio studies with mixed methods of measuring hypertension.**

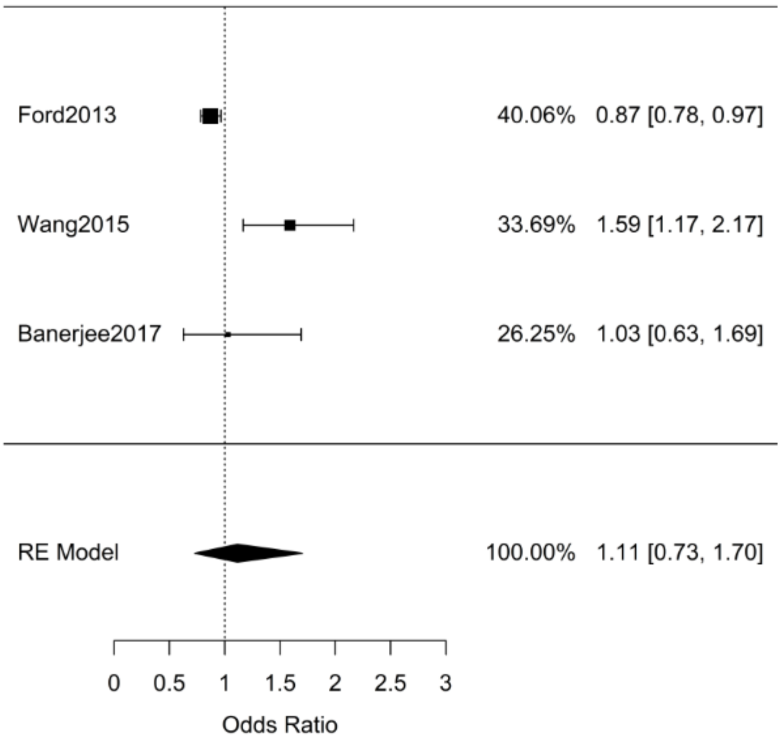

**Fig S3.3. Meta-analysis of odds ratio studies which used systolic blood pressure cutoff of 140 mm Hg to determine hypertension diagnoses.**

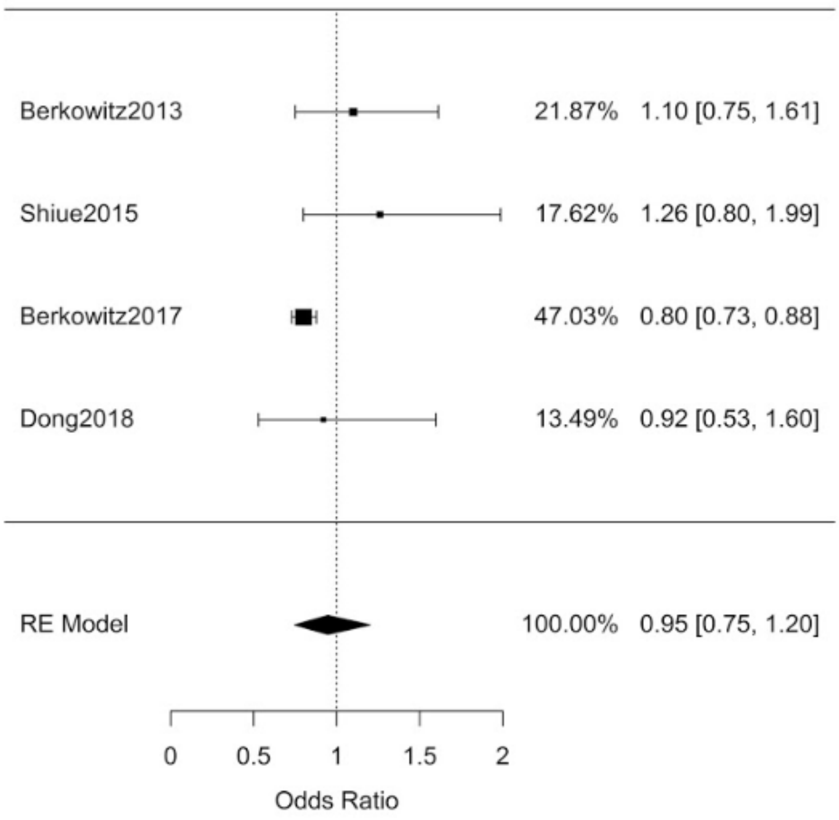

**Fig S3.4. Meta-analysis of odds ratio studies which used systolic blood pressure cutoff of 130 mm Hg to determine hypertension diagnoses.**

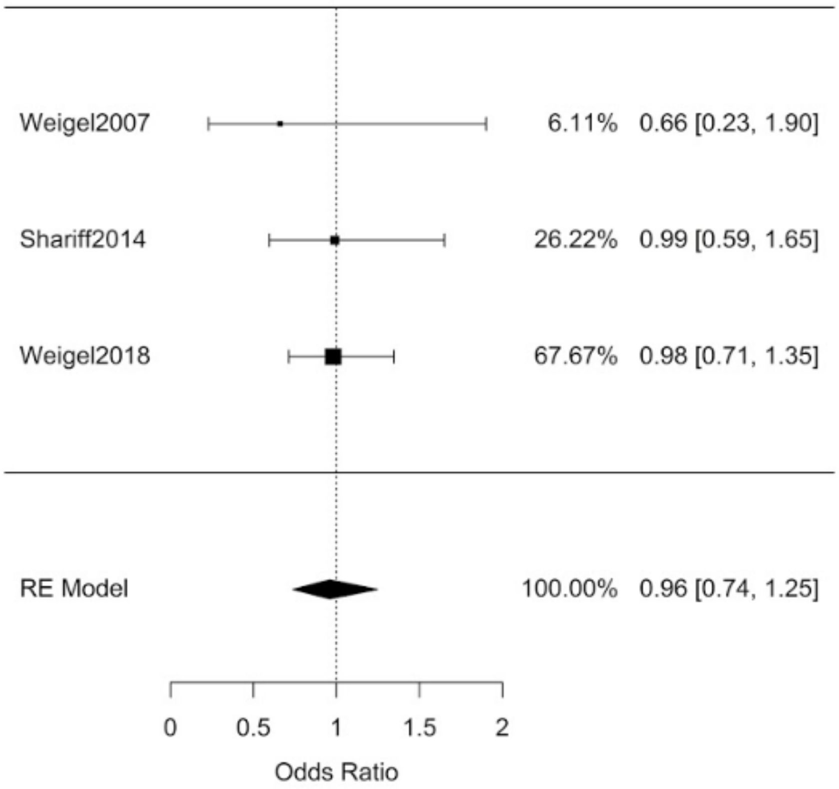

**Fig S3.5. Meta-analysis of odds ratio studies which contained data exclusive to Latinx patients.**

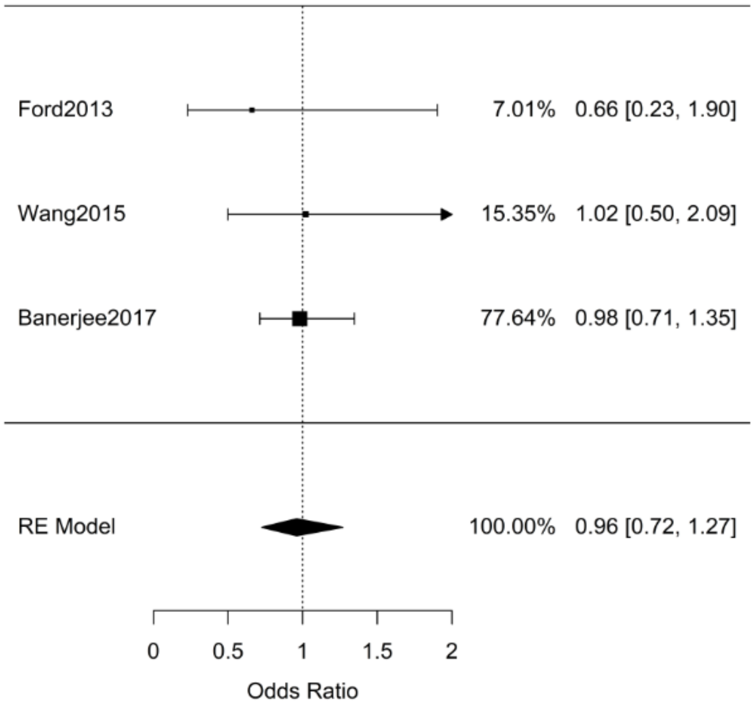

**Fig S3.6. Funnel plots and asymmetry statistical tests were performed for meta-analysis with more than 3 comparable studies.**

(A) Meta-analysis of OR (all adult studies); (B) Sub-group analysis for studies of BP measured by researchers; (C) Meta-analysis of Hedges' g of Systolic BP in adults, and (D) diastolic BP. (E) Meta-analysis of OR (all children studies). None of the funnel plot asymmetry tests were significant, although the regression test for funnel plot asymmetry was close for sub-figure B (*p* = 0.056).

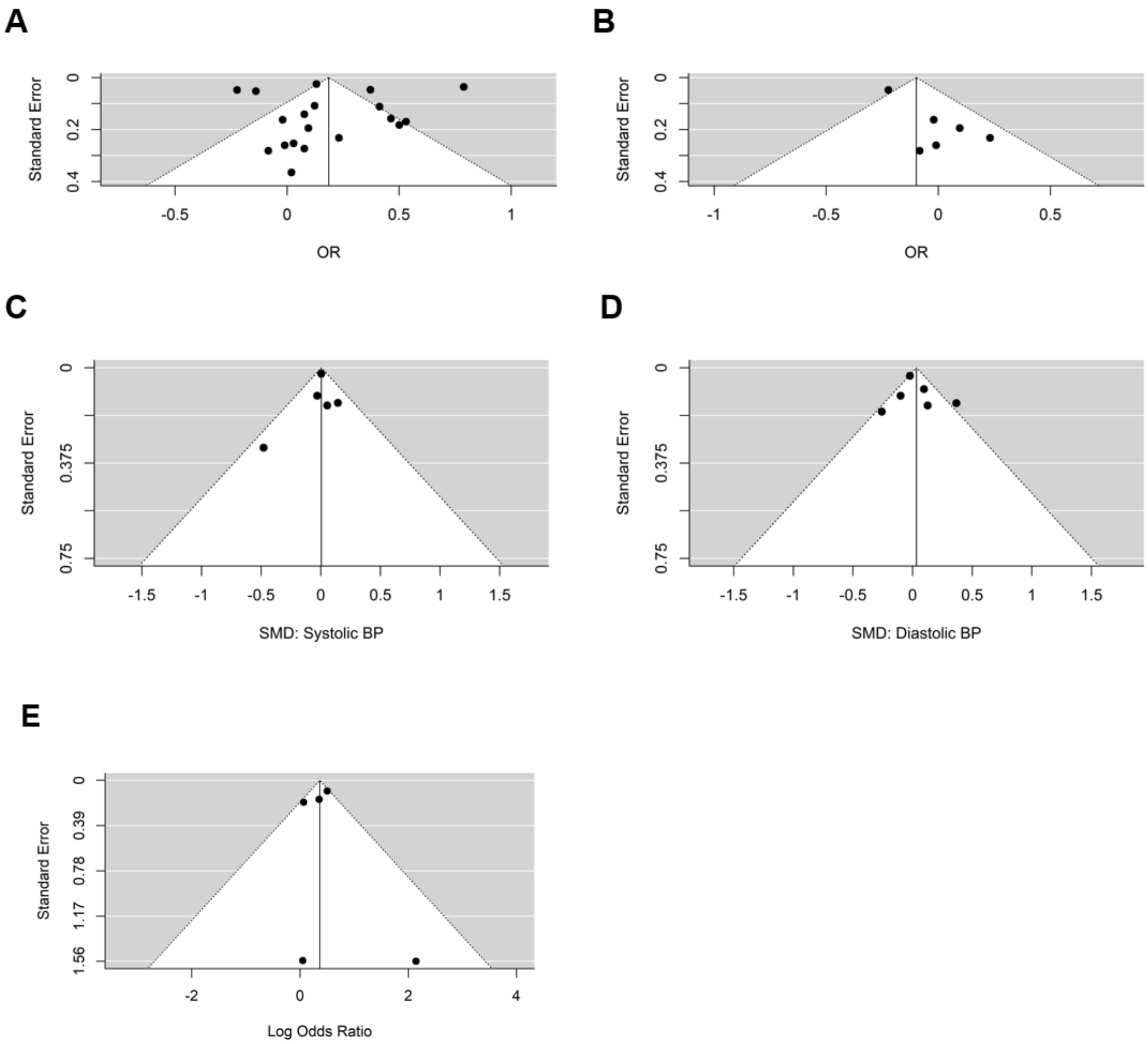

Supplement: S3 File — (PDF) [file pone.0241628.s003.pdf]
