# Supplementary material for: Food insecurity and hypertension: A systematic review and meta-analysis
Source: PLoS One. 2020 Nov 17;15(11):e0241628. doi: 10.1371/journal.pone.0241628 (PMC7671545; doi:10.1371/journal.pone.0241628)
Supplement: Attachment — Submitted filename: PONE-D-20-15615-Respone to Reviewers [9.1.20].pdf [file pone.0241628.s005.pdf]

Beltran et al
Manuscript PONE-D-20-15615
Responses to Reviewers

Reviewer #1

1. ***It should be noted that the association studied is between essential hypertension and FIS, and in this case it is necessary to eliminate any study which studies the secondary hypertension.***
   We thank the reviewer for this important point. The articles identified by this systematic review differed greatly, not only in regard to patient populations, but also in the methodology of exploring the association between FIS and hypertension. Due to insufficient studies, granularity on whether FIS causes primary and/or secondary hypertension cannot be answered in this review. Rather, we chose to group studies based on methodology and outcomes rather than particular patient populations in order for meta-analysis to be possible. To better illustrate this point, we altered the results section of the abstract to better clarify the choice to group studies based on methodology (lines 66 - 69). Furthermore, we added a brief mention of the inability of this review to assess whether food insecurity is associated with specifically primary or secondary hypertension to the limitations section (lines 439 - 442).
2. ***The conflict of interest is not mentioned in the manuscript.***
   We appreciate the reviewer's attention to this detail. A conflict of interest statement has been added to the manuscript following the conclusions section (lines 476 - 477).

Reviewer #2

1. ***Introduction needs to fulfill the criteria of the direct or predisposing relation between hypertension and food insecurity moreover***
   We agree with the importance explicitly describing the potential mechanistic link between FIS and hypertension and in fact provided this information in the final 2 paragraphs of the Introduction.

2. ***[The] definition of food insecurity needs detailed illustration.***
   We have added additional text in lines 96 - 99 of the Introduction regarding the definition of food insecurity.
3. ***No available data in the metanalysis regarding the stage of hypertension or number of medication for patients and food insecurity.***
   We thank the reviewer for this observation. We agree that specific information regarding the staging of hypertension or the use of hypertensive medications between food insecure and food secure groups is an important consideration. Unfortunately, the

studies identified in this review did not provide data related to the staging of hypertension as related to food insecurity. Furthermore, upon an additional review of the included studies, we found that no study reported data about the number of medications used by subjects. To make this point, we added this observation to the results section (lines 290 - 291 and lines 323 - 324).

4. ***Comment: Mean BMI of the group studied and association of other cardiovascular risk factors that may interfere with blood pressure control.***
   The authors agree with the reviewer's point that BMI would be an important consideration in this study. Upon re-evaluation of the included studies, we found important details regarding which studies reported BMI information or adjusted their results for subject BMI. These additional results are detailed in the results section in lines 290 - 323. As reporting of BMI data was inconsistent across included studies, this additional analysis is also mentioned in the limitations section in lines 443 - 451. Finally, as this is an important consideration in the interpretation of our results, we also added a line to mention adjustment for BMI in the abstract (lines 71 - 72).
5. ***In line 365 mentioned that majority of patients were subjected as hypertensive from a single reading which interfere with the basis of this metanalysis.***
   We agree with the reviewer's point that a single elevated blood pressure reading does not constitute a diagnosis of hypertension. We believe that this is an important limitation of several studies included in this comprehensive review. To more clearly illustrate this point, we have expanded the previous discussion on this point (see lines 436 - 439)
6. ***In line 368 patient self-reported data in measuring chronic disease also not reliable as inclusion study.***
   Similar to the previous point, we agree with the reviewer's observation regarding the unreliability of self-reported hypertension. This is an indeed a notable limitation of some of the included studies. To further clarify this point, we have added additional language in lines 452 - 456 of the limitations section.
7. ***More specific studies should be included to achieve available recommendation and conclusion.***
   We appreciate the reviewer's concern with making recommendations or conclusions based on the results of our study. As we conducted a broad initial search of the literature, it is likely that our review encompasses almost all if not all published data on the possible relationship between food insecurity and hypertension or elevated blood pressure. We have clarified this point in the abstract section, lines 55 - 56. As such, we are unable to include more specific studies which could provide a greater understanding regarding food insecurity and blood pressure or hypertension. Our review found a

largely negative association between food insecurity and hypertension or elevated blood pressure suggesting a need for further investigation into a possible relationship. As such, there are sufficient information to generate reliable recommendations at this time.
